# MINOCA: A Working Diagnosis

**DOI:** 10.7759/cureus.49695

**Published:** 2023-11-30

**Authors:** Isabel I Rodríguez Candelario, Adrian E Perez-Aybar, Jose A Roman-Ramos

**Affiliations:** 1 Cardiovascular Disease, Centro Médico Episcopal San Lucas, Ponce, PRI; 2 Internal Medicine, VA Caribbean Healthcare System, San Juan, PRI

**Keywords:** prognostic markers, coronary microvascular disease, type 2 myocardial infarction, cardiac multimodality imaging, myocardial infarction with non-obstructive coronary arteries (minoca)

## Abstract

Cases of patients presenting with myocardial infarction (MI) without angiographic obstructive CAD are not trivial and have significant prevalence. "The Fourth Universal Definition of MI" (4UDMI) published in 2018 introduced MI with non-obstructive coronary arteries (MINOCA). The new section was of great importance as it validated the diagnosis by defining its criteria and recognizing its presence in the community and the need for further investigation. Given the nature of the diagnosis of MINOCA, coronary angiography provides limited information about prognosis and risk stratification for future major adverse cardiovascular events (MACE). Thus, additional imaging to understand the underlying etiology of MINOCA in conjunction with a better understanding of prognostic factors is necessary to expand on the current guidelines and aid in screening for possible complications, risk of MACE, and all-cause mortality. Discerning the etiology of the presentation is crucial, and physiologic studies, as well as additional imaging, are an important part of this evaluation. These modalities include intravascular studies such as optical coherence tomography (OCT), intravascular ultrasound (IVUS), fractional flow reserve (FFR), and imaging in the form of cardiac CT (CCT) and cardiac MRI (CMR). This step is essential to target treatment regimens more efficiently. The purpose of promoting multiple imaging modalities beyond traditional angiography is to address the working MINOCA diagnosis, with the finality of identifying the specific ischemic pathophysiology. MINOCA has multiple causative mechanisms, making it a heterogeneous collection of etiologies, resulting in acute MI: atherosclerotic, and non-atherosclerotic. This literature revision demonstrates that MINOCA prevalence and mortality are not trivial, and the diagnosis affects quality of life. MINOCA presents a definitive risk of MACE without proper stratification and targeted medical therapy. Several prognostic factors of morbidity and mortality in MI-CAD patients have been identified to correlate with MINOCA patients, especially inflammatory markers. MINOCA is not an exclusion diagnosis but a working diagnosis for which further imaging studies should be performed.

## Introduction and background

The etiology of myocardial infarction (MI) has been studied since the beginning of the twentieth century, and the consensus on diagnosis has developed chiefly during the last two decades. The development of diagnostic catheterization aided greatly in identifying the presence of coronary thrombus or significant coronary plaque as a pathological culprit. Nevertheless, this classical clinical picture of coronary obstruction is not always found, presenting a clinical conundrum for cardiologists. The inconsistency of the angiographic findings and clinical presentations led to the need for a universal definition of MI. The publication of the Universal Definition of Myocardial Infarction, a collaboration between the European Society of Cardiology (ESC), America College of Cardiology (ACC), American Heart Association (AHA), and the World Health Organization (WHO) defined a universal diagnostic criterion and classifications of MI. In 2018, the “Fourth Universal Definition of Myocardial Infarction” (4UDMI) expanded the previous entries with new sections in which myocardial infarction with non-obstructive coronary arteries (MINOCA) was a new addition [1].

In cases of MI with the absence of obstructive coronary artery disease (CAD) in angiography, MINOCA cases, the recommendation is to pursue further imaging evaluation. Nevertheless, further research is necessary as guidelines for diagnosis and treatment are scarce and depend on additional clinician aptitude and diagnosis experience. Since the 4UDMI release, the research on MINOCA has increased, and validation of past research in traditional MI is being evaluated for MINOCA. Here, we review some of the most recent work and new study topics in MINOCA.

## Review

Universal definition

Cases of patients presenting with MI without angiographic obstructive CAD are not trivial and have significant prevalence. This type of myocardial ischemia was denominated in the community as MINOCA. The first declarations made by an academic society about this MI presentation and diagnosis were made by the European Society of Cardiology (ESC) in 2016. The term troponin-positive non-obstructive coronary arteries was coined, and it encompassed multiple diagnoses [2]. MINOCA, myocardial, and non-myocardial disorders were grouped under this term. It was not until "The Fourth Universal Definition of MI" (4UDMI) was published in 2018 that a new section was introduced: MINOCA [1]. The section was composed of 180 words in a 34-page document. Nevertheless, it was one of the most recent advances in MI categorization. The new section was of great importance as it validated the diagnosis by defining its criteria and recognizing its presence in the community and the need for further investigation.

MINOCA differs from the other more well-known MI categories by the absence of obstructing CAD in coronary angiography, in the setting of elevated biomarkers, and clinical presentation of ischemia. The obstructive lesions threshold, set at 50%, supports that thrombotic occlusion of blood supply to cardiac tissue is most likely not the aggravating factor in dysfunction and symptom presentation to cause MI. Thus, further evaluation is warranted. Following the release of the 4UDMI, the American Heart Association (AHA) published a scientific statement in 2019, "Contemporary Diagnosis and Management of Patients with Myocardial Infarction in the Absence of Obstructive Coronary Artery Disease" [3]. There, MINOCA diagnostic criteria are refined, and the exclusion of no ST-segment elevation MI (NSTEMI) type 2 and myocardial disorders is made, now being singled as separate definitions and other diagnostic entities. The diagnostic criteria for MINOCA proposed three major requirements: 1) acute MI, 2) non-obstructive coronary arteries on angiography (<50% occlusion), and 3) no specific alternate diagnosis for the clinical presentation [3].

Epidemiology

Coronary artery diseases are very well-studied conditions for which much research has resulted in significant databases. Multiple systematic reviews evaluating the epidemiological characteristics of MI patients exist, with significant concordance in results. MINOCA, a recently established diagnosis, has been studied by extrapolating data from already completed studies. Pasupathy’s systematic review explores the epidemiology of MINOCA diagnosis in 28 quantitative and 46 qualitative publications [4]. MINOCA prevalence was 5%-6% of acute MI, and most were NSTEMI with lower troponin counts and twice as prevalent in women over men of black, Hispanic, and Pacific races. Hyperlipidemia, diabetes mellitus, active smoking, and coronary family history were the most common comorbidities. These studies' strengths lie in evaluating multiple published studies of large sample sizes and power; they lack specificity in inclusion and exclusion criteria due to the heterogeneous nature of the diagnosis. Extrapolating data from prefixed databases limits the measurement of a pure MINOCA diagnosis. 

Prognosis

Given the nature of the diagnosis of MINOCA, coronary angiography provides limited information about prognosis and risk stratification for future major adverse cardiovascular events (MACE). Thus, additional imaging to understand the underlying etiology of MINOCA in conjunction with a better understanding of prognostic factors is necessary to expand on the current guidelines and aid in screening for possible complications, risk of MACE, and all-cause mortality.

A major challenge faced when analyzing existing data is the diagnosis inclusion criteria. In multiple sub-analysis studies, the MINOCA diagnosis is heterogeneous and, in some cases, includes Takotsubo and certain myocardial disorders. Differences in inclusion criteria may account for the variability and no apparent congruence of the prognosis of MINOCA in the current literature. Nevertheless, studies reveal a consensus on similar mortality and morbidity of MINOCA and MI in obstructive coronary artery disease (MI-CAD) patients.

Multiple population studies have indirectly compared MINOCA to MI-CAD regarding short- and long-term prognosis. Studies that analyze large databases, such as Smilowitz et al.’s analysis of the Acute Coronary Treatment and Intervention Outcomes Network Registry (ACTION) and Choo’s prospective study of the Korean Acute Myocardial Infarction Registry of NIH (KAMIR-NIH), have found more significant short-term mortality in MINOCA [5,6]. In contrast, Pasupathy et al.’s 2015 systematic review found in-hospital mortality more prevalent in MI-CAD [4]. This incongruence could be due to the study population, inclusion criteria, and heterogeneity of the patient database.

Long-term mortality ranged around 2% when comparing unstable angina studies CRUSADES and PURSUIT and a meta-analysis of a predictor of mortality of MINOCA patients by Pelliccia et al. [7]. The long-term impact on the health of young MINOCA patients and their recovery was observed in the VIRGO prospective study [8]. The sample in this study was representative of the general epidemiological characteristics of MINOCA patients previously described in the literature, with patients two times more likely to be women and of a young age, with a range of 18-55 years. When compared to MI-CAD patients, MINOCA patients had a lower quality of life affected by physical limitations, angina frequency, and treatment satisfaction.

The reviewed studies are based on a population level and are not specific or reflect upon the multiple etiologies within MINOCA. Prognostic indicators related to the underlying pathologic mechanisms may help stratification by prognosis.

Prognostic factors

Traditionally large cohort population retrospective studies focus on evaluating risk factors and outcomes following MI to establish MACE prognosis. Established prognostic factors in MI-CAD are being assessed in MINOCA; as these diagnoses may share underlying etiologies, it is logical to question whether the established associations hold for MINOCA. A particular interest has favored the evaluation of inflammatory markers in MINOCA, as inflammatory disruption of the endothelial state contributes to all MINOCA etiologies. Several prognostic factors will be reviewed.

Metabolic Syndrome

Metabolic syndrome is commonly presented in conjunction with MINOCA as it contributes to multiple risk factors for heart disease development. Metabolic syndrome has been found to be independently predictive of MACE in MINOCA patients, with high fasting blood sugar being the most essential factor [9].

Stress Hyperglycemia Response

Stress hyperglycemia response (SHR), a novel marker for true acute hyperglycemia, helps differentiate true hyperglycemia brought on by the inflammatory and neurohormonal response to a significant illness versus hyperglycemia presenting due to underlying metabolic dysfunction such as metabolic syndrome or diabetes. This differentiation is achieved by weighing the glucose peak level against the patient’s glycosylated hemoglobin. As studied previously, the MI population with elevated SHR has a worse prognosis than those with minimal SHR. Recent publications have studied SHR in MINOCA patients, where it was found that elevated SHR correlated with poor prognosis. The upper tertile of patients had the highest MACE hazard ratio, with a COX regression showing an association corresponding with increased MACE risk [10].

Lymphocyte and Platelet Ratios

White blood cell count to mean platelet-volume ratio (WMR) and neutrophil-to-platelet ratio (NPR) are inflammatory biomarkers that have been shown to predict clinical outcomes in patients presenting with acute MI. Mohammed et al.'s study revealed that this correlation is also present in MINOCA, and patients with high WMR and NPR were at increased risk of MACE [11]. They are pointing to the possible use of these ratios as risk prediction markers in the clinical assessment of MINOCA patients.

Increased Total Bilirubin

In patients with acute MI, elevated liver function parameters such as ALT, AST, alkaline phosphatase, and total bilirubin are related to worse outcomes, with serum total bilirubin predicting prognosis and adverse outcomes. Yin et al. demonstrated that high initial TB levels (>0.9 mg/dL) are a robust predictor of worse clinical outcomes in MINOCA patients, associated with higher MACE and rehospitalization, serving as a potential stratification tool for MINOCA patients [12].

Hyperuricemia

Hyperuricemia is an independent prognostic factor associated with poor prognosis after MINOCA. Estimating serum uric acid levels ( $\geq$420 umol/L in men or 357 umol/L in women) may facilitate risk stratification in patients with MINOCA history. Hyperuricemia was associated with a 5.9% increase in MACE with a hazard ratio of 1.498. Incorporating hyperuricemia into the TIMI score yielded significant improvement in discrimination for MACE [13].

Coronary Angiography Findings

Classifying an acute MI event as MINOCA still provides a wide range of possible findings during coronary angiography. Some patients may have no appreciable coronary artery narrowing, and others may have considerable disease not meeting the criteria for obstructive disease (<50% stenosis). In a 2023 paper in the journal *Atherosclerosis* by Tsaban et al., the authors found that non-obstructive coronary atherosclerosis is associated with worse outcomes in MINOCA versus patients with no evident atherosclerotic plaques [14]. Coronary vascular dysfunction, both at the microvasculature and epicardial level, is one of the possible causes of MINOCA presentation. MINOCA patients with coronary slow flow have a higher incidence of MACE with an adjusted HR of 2.76 vs. patients with regular coronary flow rates [15]. Additionally, the positive result of provocation testing with acetylcholine (ACh) identifies the cause of myocardial ischemia. The incidence of MACE was higher among patients with a positive ACh test (24 (13.0%) vs. 6 (4.5%), p=0.017) as an independent predictor of MACE [16]. Coronary angiography findings can contribute to a better understanding of the risk of future clinical events and help elucidate the source of the etiology of MINOCA.

Perivascular Fat Attenuation Index (pFAI)

The diagnostic algorithm of MINOCA calls for further imaging beyond the standard angiography, using intravascular imaging such as optical coherence tomography (OCT), intravascular ultrasound (IVUS), and cardiac MRI (CMR) to discern the etiology of the event. Cardiac CT (CCT) also serves as a tool that identifies the etiology while allowing for the recompilation of other prognostic factors. Peri-coronary fat inflammation, as identified by the pFAI, is a novel imaging marker of inflammation for patients who may undergo cardiac surgery. Studies have shown that it predicts outcomes in subjects with ischemic heart disease. Pergola et al. showed that patients with MINOCA had significantly higher pFAI values compared to controls when taken within eight days of the event, leading to the possibility of the use of CCT for the identification of MINOCA cases at institutions where CMR may not be available [17].

Diagnosis 

The AHA and other academic societies have designated specific criteria and a proposed sequence to establish the diagnosis of MINOCA [3]. First, a clinical presentation of ACS must be present, followed by a coronary angiographic study that reveals no obstructive lesions (<50%) to evidence non-obstructive CAD; this is when a working diagnosis of MINOCA is present. The diagnostic effort must not end here. A missed coronary obstruction or other myocardial tissue injury mechanisms must be excluded to proceed with further investigation. Multiple modalities can serve as tools in this investigation, both physiologic in nature or with imaging studies. The tools that can be utilized include intravascular studies such as optical coherence tomography (OCT), IVUS, measurements such as fractional flow reserve (FFR), and additional imaging such as CCT, and CMR. This step is crucial to target treatment regimens more efficiently.

Additional studies in MINOCA

MINOCA is a working diagnosis. Once a non-obstructive angiogram is encountered in the setting of an MI, further investigation is needed. Multiple cardiac imaging alternatives exist and should be integrated as part of the workup to identify the ischemic etiology. 

Intravascular Studies

IVUS provides a cross-sectional 360-degree vessel image. It characterizes lesion morphology and quantifies plaque burden. IVUS has been around for a more extended period than other vascular imaging techniques. OCT makes images 10 times smaller (10 um) than IVUS in 2.5 seconds. It allows for the precise evaluation of the integrity of the atherosclerosis plaque, stent sizing guidance, and expansion assessment. However, contrast is required (10-12 cc/run), and execution and interpretation expertise are needed. FFR is an invasive hemodynamic functional measure to evaluate the significance of stenosis in epicardial contrary arteries. It is the gold standard to determine if a lesion is responsible for inducing ischemia. Values under 0.75 are hemodynamically significant, and intervention is recommended. It is a class 2A recommendation in American guidelines for evaluating coronary lesions between 50% and 70% stenosis and helps guide revascularization [18]. Thus, a combination of plaque morphology and hemodynamic stenosis evaluation is essential for diagnosing MINOCA and its etiology.

CCT

Recent studies have discussed using CCT to quickly characterize plaque and inflammation in the acute stage of MI and MINOCA. CCT can also be used to evaluate the causes of MINOCA presentation. It allows for a comprehensive analysis of the coronary artery anatomy, myocardial perfusion, wall motion of ventricles, and extracardiac structures [19,20]. With the implementation of the pFAI, it is possible to use CCT for diagnostic and prognostic purposes. It promises new avenues for diagnostics in centers where CMR may not be available [17]. MINOCA-GR is a Greek population study that aimed to include CCT as a diagnostic step in the workup of patients with MINOCA in conjunction with intra-coronary angiography and CMR. In the study design, the authors discuss the benefits and diagnostic utility of CCT and prognostic scores that may be obtained through CCT [20].

CMRI

CMR studies anatomy, physiology, and pathology and is the gold standard for in-vivo detection of MI. It is a perfusion study that detects myocardial fibrosis with high diagnostic certainty, providing information on tissue viability and MI age. Multiple sequence protocols exist to feature anatomic definition and tissue characterization by detecting deoxygenated hemoglobin in tissue lesions. Myocardial enhancement studies with gadolinium contrast visualize microvascular obstruction by highlighting its abnormal diffusion pattern. This imaging is obtained with now readily available MRI machines and is a safe option for patients. An excellent tool for diagnosing tissue ischemia without epicardial coronary obstruction [19].

The purpose of promoting multiple imaging modalities beyond traditional angiography is to address the working MINOCA diagnosis, with the finality of identifying the specific ischemic pathophysiology. Opolski designed a prospective study to determine the morphology of the coronary plaques identified in MINOCA patients and asses their relationship to myocardial injury [21]. The study selected MINOCA patients and obtained OCT and CMR within seven days of coronary angiography post-MI. OCT findings were classified as plaque disruption, plaque erosion, coronary thrombus, and others. CMR findings were classified as ischemic, non-ischemic, and mixed. No association was found between plaque rupture and late gadolinium enhancement (LGE) in CMR, but a more significant frequency of LGE was found in patients with plaque rupture vs. no plaque rupture. Additionally, a higher frequency of plaque rupture was found in vessels supplying the infarct-related artery, confirmed by CMR. In this cohort, using OCT resulted in changes in intervention strategies in 16% of the cases.

This brings thought to what is to be done with the additional information the multiple imaging modalities provide. Obtaining knowledge about the specific acute pathophysiology will be essential for treatment guidance and the development of management guidelines.

Pathophysiology

MINOCA has multiple causative mechanisms, making it a heterogeneous collection of etiologies, resulting in acute MI. The AHA 2019 statement divided the causes of MINOCA into two categories: atherosclerotic and non-atherosclerotic [3]. While the atherosclerotic causes cause ischemia in a similar mechanism as MI-CAD, the non-atherosclerotic causes compose other forms of ischemia not reliant on plaque formation and subsequent embolization.

Atherosclerotic Causes

The basis of the atherosclerotic causes of MINOCA is coronary plaque disruption. Atherosclerosis causes coronary plaque formation. Plaque rupture results in a discontinuation of the fibrotic plaque creating a cavity between the plaque and the vessel lumen. Plaque erosion occurs within the continuous thrombus formation on the plaque's surface facing the vessel lumen, forming a white plaque; this causes endothelium erosion by apoptosis provoked by vasospasm. Plaque disruption occurs when plaque rupture or erosion triggers thrombus formations; this can cause inflammation, distal embolization with myonecrosis, superimposed spasm, or transient complete thrombosis with thrombolysis. These manifestations appear as haziness or minor filling defects in angiography without obstruction and can only be diagnosed by intra-coronary imaging. It is found in 1/3 of MINOCA patients examined with IVUS and in angiographically normal-appearing vessels in 50% of cases [3].

Non-atherosclerotic Causes

Non-atherosclerotic causes of MINOCA are multiple. All these etiologies center on a common mechanism, blood flow and oxygen restriction; the vessel is unable to meet the metabolic demands of the myocardial tissue. Coronary artery vasospasm (CAS) can occur at the level of the epicardial arteries and inside the microcirculation. This etiology is complicated to detect because it requires intraluminal measurements during an event; this can be triggered by intracoronary acetylcholine (ACh) and detected by coronary flow reserve measurement or microvascular resistance [16]. Underlying mechanisms for CAS are varied and numerous but center around endothelial dysfunction. Spontaneous coronary artery dissection (SCAD), a separation of the layers of the epicardial coronary artery, is an underdiagnosed etiology. The hemorrhage that results from the dissection may progress to cause complete occlusion of the vessel resulting in ischemia. Coronary artery embolism (CE) may originate from sites such as the left atrial appendage, heart valves, or coronary arteries. Thromboembolism can also be caused iatrogenically or secondary to coagulation disorders. Coronary microvascular dysfunction (CMD) is a cause of myocardial ischemia that affects the small vessels that receive the coronary outflow. This dysfunction is only diagnostically feasible by intracoronary flow measurements through the epicardial vessels. Calculations such as the index of microcirculatory resistance (IMR) can point to microvascular dysfunction [22]. Supply-demand mismatch occurs in extreme scenarios where non-obstructive plaque reduces the epicardial vessels' oxygen-transferring capacity, leading to insufficient oxygen delivery to the myocardium. When the heart is stressed sufficiently, the demand for perfusion can become more significant than ever without obstruction ischemia.

Medical therapy

Classic coronary medical therapy is directed toward atherosclerotic burden control, which is reduced in MINOCA patients. Coronary artery disease Class I recommendations about secondary prevention medical treatment, risk factor modification, and cardiac rehabilitation exist for MI-CAD patients but none for MINOCA patients. MINOCA treatment guidelines should be developed by evidence-based medicine considering the diverse ischemic etiologies. There are no existing results of randomized control studies evaluating the effects of pharmacotherapy in MINOCA patients. MINOCA-BAT is the first clinical trial designed to analyze a goal of 3,500 patients in a case-control ACE-inhibitor/angiotensin receptor blocker (ACE/ARB) vs. placebo therapy for MACE. This study has not been completed yet [23]. Currently, medical treatment is guided by extrapolated data from observational studies.

Lindahl et al. created a sub-study from the SWEDEHEART registry, which includes over 200,00 patients with MI diagnosis. They analyzed over 9,000 MINOCA patients with a four-year follow-up to evaluate the relationship between classic coronary preventive disease treatment and mortality [24]; this is the first study evaluating this relationship. Results reported a significant benefit of using statins and ACE/ARB for MINOCA mortality. Statins aid in delaying the progression of atherosclerosis and have protective effects on the endothelium. At the same time, ACE helps with pressure control and endothelial function and prevents heart remodeling, all of which help ischemic myocardium. A positive trend with the use of beta-blockers (BB) and dual antiplatelet therapy (DAPT) for mortality was also recognized. For BBs, international guidelines do not recommend their use during acute MI but agree with their use for cardiac dysfunction. Reducing sympathomimetic activity that stresses the heart benefits dysfunctional myocardial tissue, regardless of the underlying etiology. The was a failure of association for mortality reduction with DAPT, questioning the theory of transitory thrombus as an MI cause. These data are consistent with other literature and support using cardioprotective medications in MINOCA patients.

Since the etiology of MINOCA is varied, the ideal medical therapy would be one tailored to the underlying pathology. This is the main reason why the diagnostic evaluation and determination of the etiology is an essential step in treatment. Cardioprotective treatment following the acute MI guidelines should be adopted for atherosclerotic causes where plaque disruption is the causative mechanism of MINOCA [18]. For CAS, the cornerstone of therapy is using calcium channel blockers (CCB) and nitrates [25]. In the case of CMD, CCB, BB, and statins are the mainstay of treatment, but due to multiple etiologies, cases should be individualized [22,26]. There is still much debate about the correct course of action in SCAD. However, BBs alleviate the stress on the epicardial vessels and diminish myocardial demand, helping control symptoms and prevent progression [27,28]. In the case of coronary thromboembolism (CE), a hematology specialist should be involved from early in work, and the use of anticoagulants and antiplatelet therapy is the mainstay for coagulopathies. For supply demand cases, the reversal of the cause of extreme demands of the heart should be reversed as quickly and safely as possible [3].

Medical treatment for MINOCA patients needs to be reinforced to practitioners, as these patients, on average, are prescribed less medication on discharge after their first event [24]. It is important to create awareness of the diagnosis, morbidity, and mortality of MINOCA and the importance of pursuing etiology identification to tailor therapy as best as possible.

Future direction

MINOCA is a working diagnosis that, when encountered, needs further analysis to identify the ischemic etiology. Additional studies are needed to validate the routine performance of OCT, IVUS, CMR, and CCTA to define the ischemic etiology; safety and cost-effectiveness must be analyzed for benefit. Evaluation of etiology-directed medical therapy is needed, for which the MINOCA-BAT study is expected to provide some results in 2025 [23]. Prognostic factors that have been validated in traditional MI should continue to be evaluated for MINOCA patients. Additionally, an ICD10 codification for MINOCA is needed to simplify future database studies and signal to providers and healthcare teams that further testing or imaging is required. This novel classification for MI has many unanswered questions for which continued investigation and innovation are needed.

**Figure 1:**
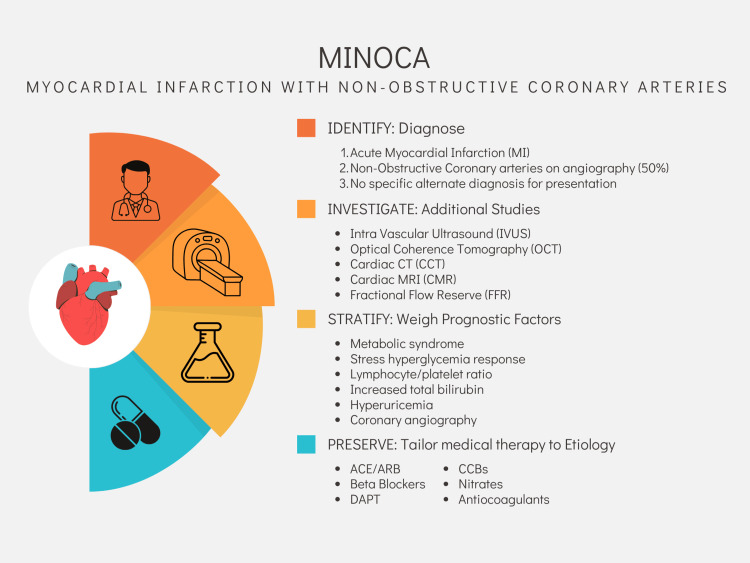
Identifying a MINOCA presentation and tailoring medical therapy to the underlying etiology.

For the general practitioner, MINOCA may be a confusing presentation as some of the steps needed to complete the workup of the MINOCA etiology might be done on an outpatient basis and may require input from primary care providers. Because of this, it is important that these physicians understand the full work-up cycle and the multiple etiologies that can cause an MI that are not occlusive in nature. Knowledge of how to continue the investigation of the causative etiology can become a shared responsibility between cardiologists and general practitioners and having a good understanding of the condition will benefit the patient. This will not only allow a better selection of medical therapy but also prompt referral to the correct imaging center or specialist for continued evaluation. In Figure 1, we summarize a four-step strategy to arrive at the correct etiology of MINOCA events and how this ultimately allows the practitioner to tailor medical therapy against the underlying pathologic mechanism of the event.

## Conclusions

This literature review demonstrates that MINOCA prevalence and mortality are not trivial, and the diagnosis affects quality of life. MINOCA presents a definitive risk of MACE without proper stratification and targeted medical therapy. Several prognostic factors of morbidity and mortality in MI-CAD patients have been identified to correlate with MINOCA patients, especially inflammatory markers. MINOCA is not an exclusion diagnosis, but a working diagnosis for which further imaging studies should be performed. Additional diagnostic tests beyond angiography are recommended, as non-obstructive coronary atherosclerosis is an underestimated and unattended cause of MINOCA. Multimodality imaging that may provide additional information are OCT, IVUS, FFR, CMR, and CCTA. Determining the pathophysiology behind a MINOCA presentation is challenging but necessary to establish an optimal management strategy. Because of this, the development of a consensus diagnostic algorithm to aid practitioners in management is essential. Physicians should recognize the mortality of MINOCA, preserve cardioprotective therapy in their medication reconciliation, and continue the workup of patients until causative etiology is known.

For the general practitioner, it is imperative that they hold sufficient knowledge of the multiple possible etiologies and how to continue the workup of the disease outside of the hospital but also work in collaboration with cardiologists in selecting the correct empiric or tailored medical therapy for each individual patient's presentation. This approach can be summarized in four simultaneous steps, identification of a MINOCA event, investigation of possible etiologies with additional studies and procedures, stratifying for the patient's personal risk factors, and tailoring medical therapy to the unique pathology.
